# A new role for anandamide: defective link between the systemic and skin endocannabinoid systems in hypertrophic human wound healing

**DOI:** 10.1038/s41598-020-68058-3

**Published:** 2020-07-07

**Authors:** Inês B. Correia-Sá, Cláudia M. Carvalho, Paula V. Serrão, Ana I. Loureiro, Carlos Fernandes-Lopes, Marisa Marques, Maria A. Vieira-Coelho

**Affiliations:** 1 Department of Plastic, Reconstructive and Aesthetic Surgery and Burn Unit, Faculty of Medicine, University of Porto and Centro Hospitalar São João, EPE, Al. Prof. Hernâni Monteiro, 4200 – 319 Porto, Portugal; 2 Department of Biomedicine - Pharmacology and Therapeutics Unit, Faculty of Medicine, University of Porto, Porto, Portugal; 3 Laboratory of Pharmacology, Department of Research and Development, BIAL-Portela & Cª., S.A., Trofa, Portugal; 4 MedInUP–Centro de Investigação Farmacológica E Inovação Medicamentosa, Universidade do Porto, Porto, Portugal

**Keywords:** Physiology, Medical research

## Abstract

The use of cannabinoids to treat fibrotic skin diseases is an emergent issue. Therefore, we aimed to evaluate systemic and skin endocannabinoid responses in the wound-healing process in humans. A prospective study was performed in 50 patients who underwent body-contouring surgery. Anandamide (*N*-arachidonoylethanolamine, AEA), 2-arachidonoylglycerol (2-AG), palmitoylethanolamide (PEA) and oleoylethanolamide (OEA) were quantified using LC–MS/MS. Ten (20%) patients developed hypertrophic (HT) scars. No significant changes were observed between the normal (N) scar and HT scar groups in terms of plasma and skin endocannabinoids. Nevertheless, a positive correlation between plasma and skin AEA concentrations was found in the N group (r = 0.38, *p* = 0.015), which was absent in the HT group. Moreover, the AEA concentration was significantly lower in HT scar tissue than in normal scar tissue (0.77 ± 0.12 ng/g vs 1.15 ± 0.15 ng/g, *p* < 0.001). Interestingly, in all patients, the surgical intervention produced a time-dependent effect with a U shape for AEA, PEA and OEA plasma concentrations. In contrast, 2-AG plasma concentrations increased 5 days after surgery and were reduced and stabilized 3 months later. These results suggest crosstalk between systemic and local skin endocannabinoid systems during human wound healing. AEA appears to be the most likely candidate for this link, which is deficient in patients with HT scars.

## Introduction

Endocannabinoids are the endogenous ligands for cannabinoid receptors CB1 and CB2, which are two G-protein coupled receptors that have a widespread distribution throughout the body^1,2^. The most studied endocannabinoids are the arachidonic acid derivatives N-arachidonoylethanolamine (AEA)^3^ and 2-arachidonoylglycerol (2-AG)^4^. Palmitoylethanolamide (PEA) and oleoylethanolamide (OEA) are N-acylethanolamines (NAEs) that act by influencing AEA metabolism and binding to peroxisome proliferator-activated receptor alpha (PPAR-α) and to transient receptor potential cation channel subfamily V member 1 (TRPV1)^5–7^. Endocannabinoids and related NAEs play an essential role in many physiological central and peripheral processes. These include emotional responses, cognition, memory, motor behaviour, immune function, feeding, energy consumption and metabolic regulation at the systemic and cellular levels^8–13^.

Endocannabinoids are present in human blood, and their concentrations are dynamic. Food consumption, obesity, exercise, sleep pattern, time of the day, stress, anxiety, inflammation and pain are known to modify the endocannabinoid concentrations in the circulation^14^. They have also been quantified in other biological samples obtained from humans, including saliva^15^, hair^16^, semen^17^, breast milk, and amniotic fluid^18^.

In the skin, the endocannabinoid system has been identified in epidermal keratinocytes, melanocytes, mast cells, fibroblasts, sebocytes, sweat gland cells and hair follicle cells^19–25^. Here, it is involved in a large number of biological processes, such as proliferation, growth, differentiation and survival, immunocompetence, tolerance^26^ and melanogenesis^27^. It was recently shown that abuse of synthetic cannabinoids can result in dermatologic disorders, such as premature skin ageing, hair loss and greying, or acne^28^, indicating that cannabinoid signalling can influence skin biology.

In fact, some authors have proposed a new “C(ut)annabinoid” system^29^. This “C(ut)annabinoid” system has been linked to skin fibrosis and wound healing in animals. CB2 selective agonists and CB1 selective antagonists significantly decrease subcutis inflammatory cell infiltration (T cells and macrophages), fibroblast activation and experimental fibrosis in bleomycin-challenged mice^30,31^. It has also been demonstrated in a murine model that skin incisions produce dynamic alterations in the expression pattern of CB1^32^ and CB2^33^ receptors during wound healing in various immune cells as well as in fibroblasts/myofibroblasts. Regarding the clinical efficiency of cannabinoids in human skin fibrotic diseases, only scarce evidence is available. A short literature report including three patients exhibiting epidermolysis bullosa described faster wound healing following the self-administration of cannabidiol (CBD)^34^. Recently, a small clinical study described a beneficial effect of topical cannabidiol in acne scars^35^.

Medical cannabis is now legal in several countries. In addition, persuasive advertisement for cannabis products, namely, for dermatological treatments, and easy availability have led to an increase in consumption. In contrast, knowledge concerning the role of the endocannabinoid system in the pathophysiological responses in human skin fibrosis is missing.

In view of this fact, we quantified the most extensively studied endocannabinoids, including AEA, 2-AG and related NAEs, OEA and PEA, during different phases of wound healing in patients who later developed normal and hypertrophic scars. Our specific aims were to (1) quantify endocannabinoids and related NAEs in skin and in scars; (2) identify differences in the concentration of endocannabinoids and related NAEs in plasma and skin in humans with normal and hypertrophic scars; (3) identify potential fluctuations in endocannabinoid and related NAEs concentrations in plasma before surgery and during the different phases of wound healing (inflammatory, proliferative and remodelling phases); and (4) identify correlations between the concentration of endocannabinoids and related NAEs found in plasma and in skin from the same patient.

## Results

### Patient characteristics

All the patients included in the study were female. The mean age was 43 ± 11 (20–65) years. The mean body mass index (BMI) was 27.38 ± 3.45 kg/m^2^, and nineteen (38%) patients had previously undergone bariatric surgery. There were no significant differences in age and BMI between the two groups. Twelve (24%) patients reported smoking habits, but none reported alcohol or other drug abuse. The results are presented in Table 1.

**Table 1:** Patient characteristics at baseline in subjects who later developed normal scars (N) or hypertrophic scars (HT).

	N	HT	Total
No. of women, n (%)	40 (80%)	10 (20%)	50 (100%)
No. of surgeries, n	49	13	62
Age in years, mean ± SD	43 ± 11	43 ± 11	43 ± 11
Body mass index; mean ± SD	27.21 ± 3.43	28.05 ± 3.47	27.38 ± 3.45
Smoking, n (%)	11 (27.5%)	1 (10%)	12 (24%)
Bariatric surgery, n (%)	16 (40%)	3 (30%)	19 (38%)

In total, 40 abdominoplasties, 5 arm lifts and 5 thigh lifts were performed. Of the patients submitted to an abdominoplasty, 32 developed N scars, and 8 developed HT scars. Of the patients who underwent arm lifts and thigh lifts, 4 developed normal scars, and 1 developed HT scars after each surgery. The concentrations of endocannabinoids and related NAEs from skin collected from these different locations were compared, and no differences were found (data not shown). As a result, all the collected samples were studied together.

### Quantification of endocannabinoids and related NAEs in skin and scar samples

The concentrations of endocannabinoids and related NAEs in human skin samples collected during body-contouring surgery at time 0 are listed in Table 2. Six months after surgery, all patients were reviewed by two plastic surgeons for scar classification^36^. Patients were then classified into two different groups: those who developed normal scars (N group, n = 40) and those who developed hypertrophic scars (HT group, n = 10).

**Table 2:** Concentration of endocannabinoids (AEA, 2-AG) and related NAEs (PEA, OEA) in human skin samples collected during body-contouring surgery (t0).

	n	AEA (ng/g)	PEA (ng/g)	OEA (ng/g)	2-AG (ng/g)
N	40	1.05 ± 0.06	22.81 ± 2.49	27.73 ± 2.45	115.75 ± 13.75
HT	10	1.30 ± 0.15	23.31 ± 4.41	23.31 ± 5.06	140.64 ± 39.29
Total	50	1.09 ± 0.05	22.90 ± 2.16	27.98 ± 2.18	120.82 ± 13.24

Later, 40 patients exhibited a normal healing process (N), and 10 patients developed a hypertrophic scar (HT).

No significant differences were observed between the N and HT groups for all the endocannabinoids and related NAEs quantified in the skin collected at the time of surgery, namely, AEA, PEA, OEA and 2-AG. Large individual variability was observed for PEA, OEA and 2-AG, contributing to the high SEM values in some groups. 2-AG was the most abundant endocannabinoid found in human skin (120.82 ± 13.24 ng/g), with concentrations 119-fold higher than that of AEA (1.09 ± 0.05 ng/g, *p* < 0.001; ratio 2-AG/AEA in skin: 118.50 ± 13.69), sevenfold higher than that of PEA (22.90 ± 2.16 ng/g, *p* < 0.001; ratio 2-AG/PEA in skin: 6.88 ± 0.75) and fivefold higher than that of OEA (27.98 ± 2.18 ng/g, *p* < 0.001, ratio 2-AG/OEA in skin: 5.16 ± 0.56).

In the end of the study protocol, since bariatric patients are frequently submitted to several surgeries (see Table 1), we had the opportunity to collect a small amount of the original scar in 12 patients (N group: n = 9; HT group: n = 3), to further study the endocannabinoid system in the scar.

Considering these 12 patients, we evaluated the individual paired changes from time 0 (skin) to time 1 (scar). Endocannabinoids and related NAEs present in scar tissue were in the same range as those found in the normal skin, indicating a full and active cannabinoid function in the scar tissue (data not shown). However, no other conclusions were obtained, since we had only a low number of scars included in each group.

**Figure 1:**
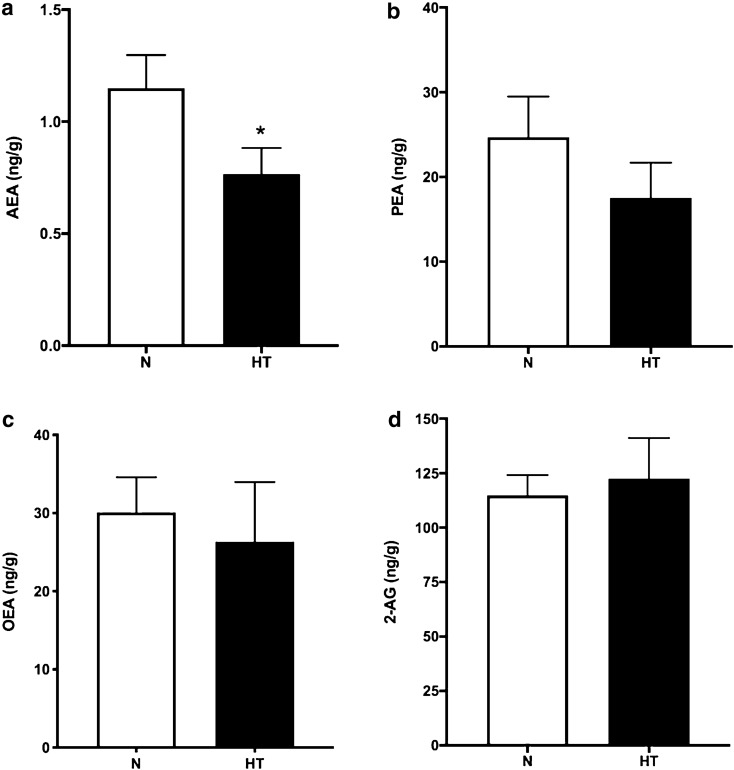
Concentration of endocannabinoids and related NAEs in scars. AEA (**a**), PEA (**b**), OEA (**c**), 2-AG (**d**) in normal scars (N, n = 15) and hypertrophic scars (HT, n = 10). *p < 0.05.

Taking these results into account, we decided to collect scars from other patients who did not pertain to this protocol and were undergoing scar correction surgery. In this group, we also classified the scars as normal or hypertrophic^36^. Table 3 resumes demographic data regarding those 25 patients. All of the included patients had their scar-inducing surgery within one year before the scar correction surgery. The period of time elapsed since the scar-inducing surgery and scar sample extraction was not different between the N and HT groups. In total, we concluded our study with 15 normal scars and 10 hypertrophic scars. In this larger sample, the AEA concentration was lower in HT scars than in normal scars (0.77 ± 0.12 ng/g vs 1.15 ± 0.15 ng/g, respectively, *p* < 0.001; unpaired t-test; Fig. 1a). There were no significant changes in PEA, OEA, or 2-AG between normal and hypertrophic scars (Fig. 1b–d).

**Table 3:** Patients characteristics that underwent scars correction surgery with normal (N) and hypertrophic (HT) scars.

	N	HT	Total
No. of women, n (%)	15 (60%)	10 (40%)	25 (100%)
Age in years, mean ± SD	49 ± 10	52 ± 17	50 ± 13
Body mass index; mean ± SD	27.78 ± 3.74	29.13 ± 4.14	28.38 ± 4.45
Smoking, n (%)	4 (27%)	1 (10%)	5 (20%)
Bariatric surgery, n (%)	7 (47%)	1 (10%)	8 (32%)

### Quantification of endocannabinoids and related NAEs in plasma

Before surgery, concentration of endocannabinoids (AEA and 2-AG) and related NAEs (PEA and OEA) in plasma were similar between patients who developed N scars and those who developed HT scars (Table 4).

**Table 4:** Concentration of endocannabinoids and related NAEs in plasma collected immediately before surgery (time 0), in patients who later exhibited a normal healing process (N, n = 40) and in patients who developed a hypertrophic scar (HT, n = 10).

	n	AEA (pg/mL)	PEA (pg/mL)	OEA (pg/mL)	2-AG (pg/mL)
N	40	950 ± 48	2,839 ± 164	2,954 ± 155	3,763 ± 378
HT	10	961 ± 155	2,971 ± 376	3,049 ± 357	4,401 ± 1,401
Total	50	952 ± 49	2,865 ± 150	2,973 ± 141	3,891 ± 404

Regarding the relative abundance, the endocannabinoids and related NAEs followed the same profile in skin and in plasma: 2-AG > OEA = PEA > AEA (*p* < 0.001). However, the ratio between 2-AG and the other compounds differed between skin and plasma. 2-AG was 119-fold higher than AEA in skin but only fourfold higher in plasma (118.50 ± 13.69 vs 4.08 ± 0.35, *p* < 0.001).

**Figure 2:**
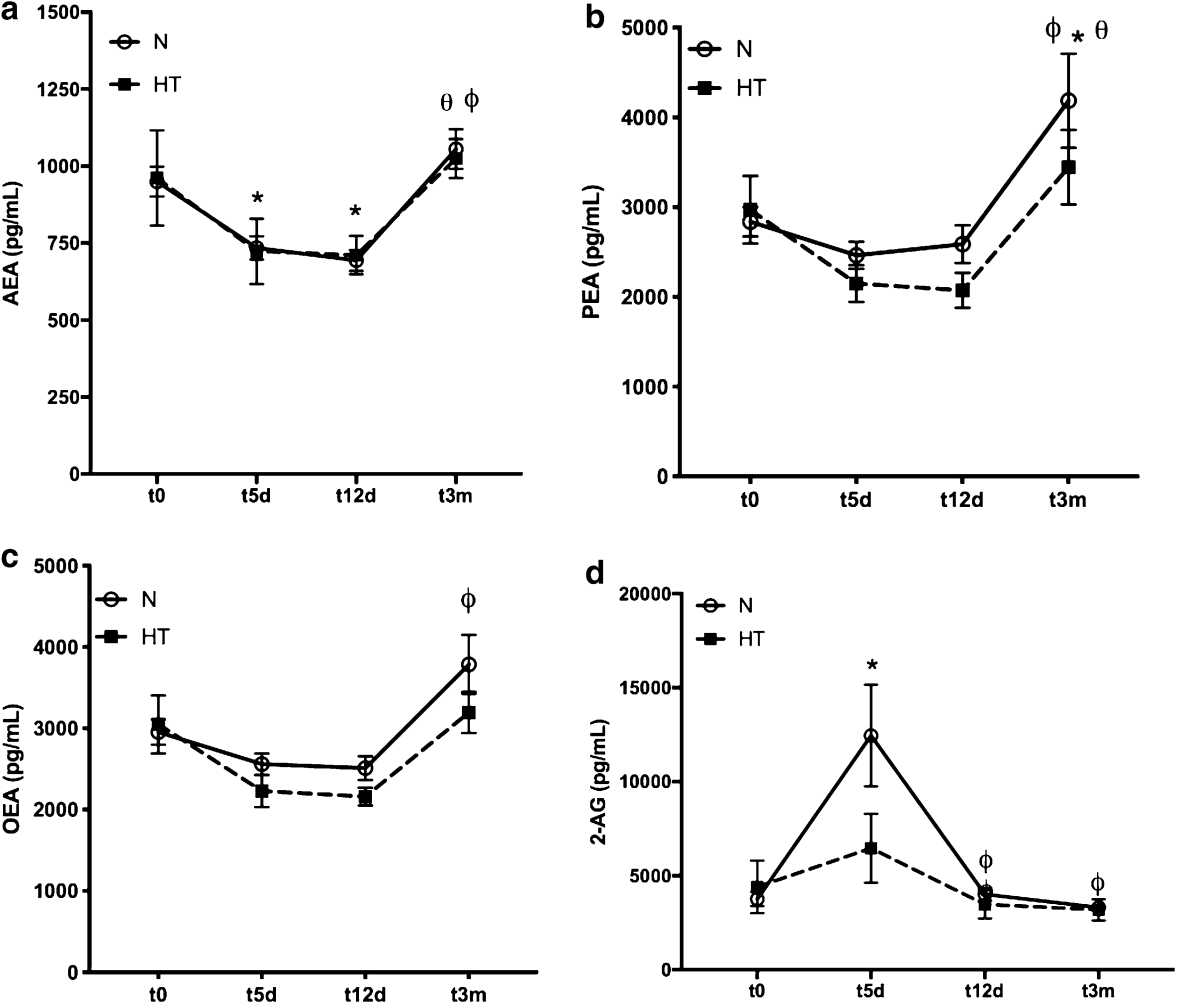
Concentration of endocannabinoids and related NAEs in plasma. AEA (**a**), PEA (**b**), OEA (**c**), 2-AG (**d**) in patients who developed normal scars (N, n = 40) and patients who developed hypertrophic scars (HT, n = 10). Samples were collected immediately before surgery (t0), 5 days after surgery (t5d), 12 days after surgery (t12d) and 3 months after surgery (t3m). Four plasma samples for each patient were collected at different times. *p < 0.05 comparing t0 values in the control group; Φ *p* < 0.05 comparing t5d values. Θ Comparing t12d values (Tukey’s multiple comparison test).

As shown in Fig. 2, no significant differences were observed in endocannabinoid and related NAE concentrations between the N and HT groups at any time after surgery. However, it was clear that a time-dependent effect along with a U shape was found for AEA, PEA and OEA (Fig. 2a–c). This profile was more evident in AEA concentrations, with a significant and sequential decrease at 5 and 12 days after surgery (956 ± 9 pg/mL vs 729 ± 8 pg/mL, *p* = 0.003 and 702 ± 12 pg/mL, *p* < 0.001) and a significant increase 3 months after surgery (1,040 ± 22 pg/mL, *p* < 0.001). In contrast, 2-AG concentrations significantly increased 5 days after surgery (3,891 ± 404 pg/mL vs 11,194 ± 2,193 pg/mL, *p* = 0.023) and became significantly lower and stabilized 12 days after surgery (3,882 ± 306 pg/mL, *p* = 0.009), reaching the lowest concentrations at 3 months after surgery (3,289 ± 265 pg/mL, *p* = 0.003).

### Relationship between endocannabinoids and related NAEs in plasma and in skin

**Figure 3:**
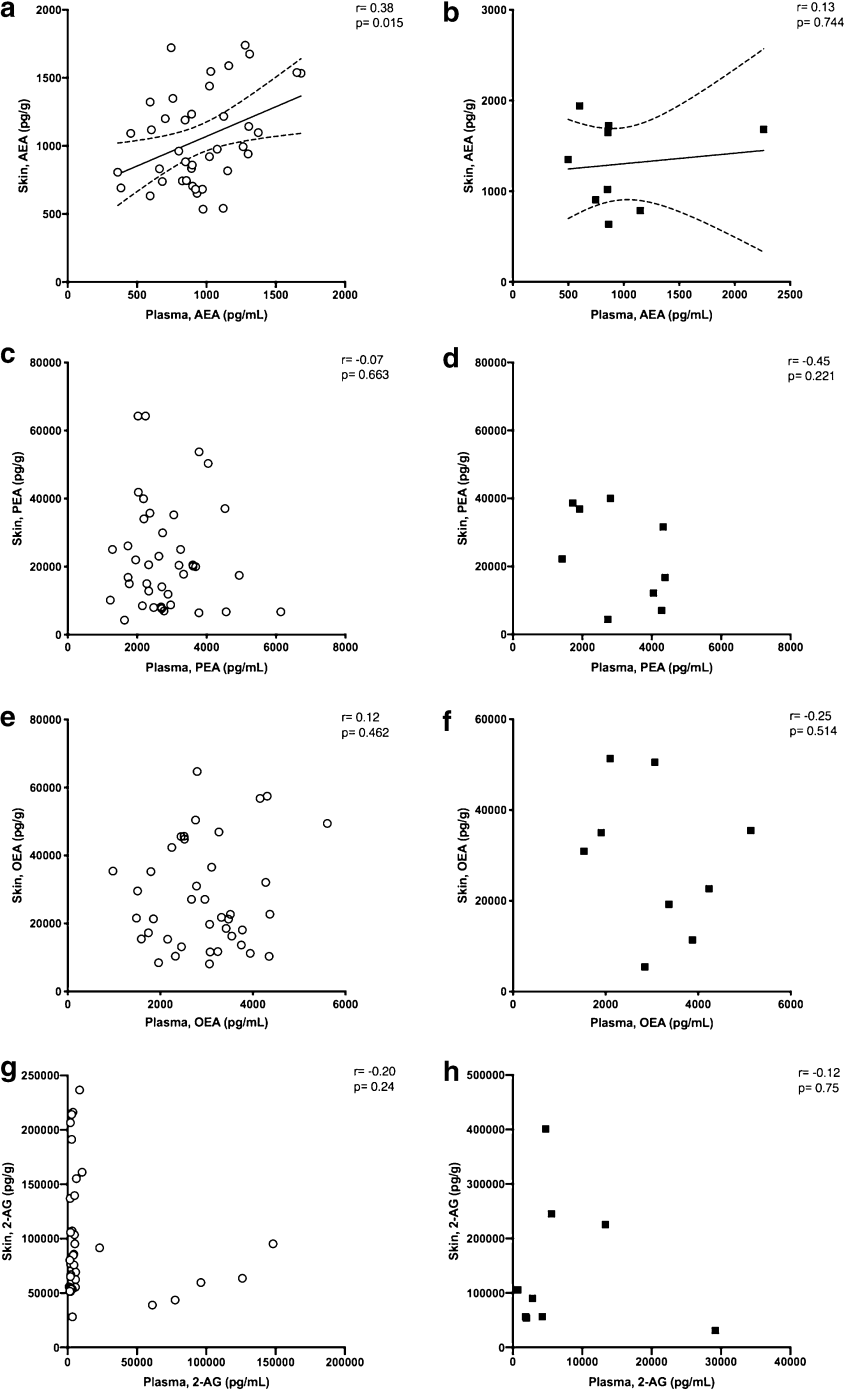
Concentration of endocannabinoids and related NAEs in plasma and skin. Relationship between the concentrations of AEA in (**a**) normal scars and (**b**) hypertrophic scars; PEA in (**c**) normal scars and (**d**) hypertrophic scars; OEA in (**e**) normal scars and (**f**) hypertrophic scars; and 2-AG in (**g**) normal scars and (**h**) hypertrophic scars in the plasma and skin of patients who developed normal and hypertrophic scars. A positive correlation with a Pearson r of 0.38 (*p* = 0.0152) with a linear regression slope of 0.44 ± 0.17, including a 95% confidence limit, was found for AEA in patients who developed normal scars (**a**). No significant correlation was found for AEA in patients who developed HT scars (**b**). No significant correlation was found for PEA (**c**, **d**), OEA (**e**, **f**), and 2-AG (**g**, **h**) in either group of patients.

To investigate a possible association between the systemic and local skin endocannabinoid systems, we tried to find a correlation between the concentrations of the endocannabinoids and related NAEs measured in plasma and in skin for each patient. At time 0, we collected plasma and skin samples from each patient. In Fig. 3a, b, we show the results for AEA in patients who developed normal and hypertrophic scars. A positive correlation between the concentrations of AEA in plasma and in skin with a Pearson r of 0.38 (a significant *p* = 0.015) was found. A linear regression with a slope of 0.44 ± 0.17 is shown in Fig. 3a, including a 95% confidence limit. In contrast, this correlation was lost in patients who developed hypertrophic scars (Fig. 3b), where the Pearson r was 0.13 (not significant). Concerning PEA (Fig. 3c, d), OEA (Fig. 3e, f) and 2-AG (Fig. 3 g, h), no significant correlation was found between plasma and skin endocannabinoid concentrations for normal or hypertrophic patients.

## Discussion

Wound healing has been conceptually divided into three distinct phases: inflammation, proliferation and remodelling. Different cells and cytokines are involved in each wound-healing phase^37^. The endocannabinoid system has been recently implicated in wound healing and skin fibrosis in mice^30,31^. Despite the growing interest in this topic, its role in human wound healing has not yet been described. Therefore, this is the first study measuring endocannabinoids in skin in the context of surgery and wound healing. With two plastic surgeons in our team, we were able to collect skin from patients, aiming to quantify endocannabinoids and related NAEs in human skin and scars. Concerning the basal concentrations of endocannabinoids and related NAEs in skin, no significant differences were observed between the N and HT groups. We also found that 2-AG is the most abundant endocannabinoid in human skin, with concentrations 119-fold higher than that of AEA, 7-fold higher than that of PEA and 5-fold higher than that of OEA. This is the first time that endocannabinoids and related NAEs have been quantified in full-thickness human skin, so we cannot compare our findings with those of previous studies; however, the AEA concentrations in these tissues are in the same range of AEA concentrations reported in human hair^38^. The relative proportion of endocannabinoids and related NAEs that we observed in human skin (2-AG > OEA = PEA > AEA) is similar to reported data obtained from different human tissues, namely, the uterus and plasma^39,40^, but different ratios have also been described in the liver^41^ and plasma^42^. Interestingly, the concentrations of the studied endocannabinoids and related NAEs in scar tissue are in the same range as those found in normal skin, demonstrating the presence of these active molecules in scar tissue. We also found that AEA is significantly reduced in hypertrophic scars compared to normal scars. This finding supports the hypothesis of a significant role for AEA in the pathophysiological process of skin fibrosis. Pathologically excessive dermal fibrosis and aberrant scarring characterize hypertrophic scars. Although the exact pathogenesis and aetiology are still unsettled^43^, it is believed that a sustained inflammatory phase is an essential prerequisite for this disorder, with a decrease in apoptosis and an increase in inflammation^44,45^. Endocannabinoids can regulate immune function and are generally considered to be anti-inflammatory agents^46^. Fatty acid amide hydrolase (FAAH) inhibitors have been proven to inhibit lipoteichoic acid (LTA)-induced pro-inflammatory responses in a CB1 and CB2 receptor-dependent manner. Topical application of a FAAH inhibitor reduced dust mite-induced skin inflammation in NC/Tnd mice with the same efficiency as the positive control tacrolimus^47^. FAAH is responsible for AEA and other NAE metabolism, and its inhibition locally increases this endocannabinoid concentration^48^. Moreover, AEA was recently shown to suppress the production and release of key Th1- and Th17-polarizing cytokines (IL-12 and IL-23) via CB1-mediated inhibition of mammalian target of rapamycin (mTOR) in human keratinocytes^49^. We suggest that reduced AEA in hypertrophic scars may be related to increased inflammation or a prolonged inflammatory phase that predisposes patients to this condition. It would be interesting to measure FAAH activity in both hypertrophic and normal scars. More studies are needed to confirm this hypothesis, but if corroborated, the topical administration of AEA, other non-psychotropic cannabinoids or FAAH inhibitors could be an interesting tool to treat or prevent this condition.

In addition to skin tissue and scars, we also had the opportunity to collect blood samples from all patients before and after wounding at three different time points, corresponding to the different phases of wound healing: 5 days for inflammation (t5d), 12 days for proliferation (t12d) and 3 months after surgery/wounding for remodelling (t3m). This allowed us to take a glimpse on what is happening in circulatory endocannabinoids and related NAEs in the four perioperative periods. Although no differences were observed at any time between the two different studied groups (N or HT group), a systematic fluctuation pattern in the concentration of all the endocannabinoids and related NAEs was observed. Interestingly, while AEA and NAEs presented a U shape after surgery, characterized by significantly lower concentrations in circulation at t5d and t12d and with the normal concentration restored at t3m, 2-AG showed a completely different pattern, with an increased concentration at t5d and a progressive decrease at t12d and t3m. It is not surprising that different endocannabinoids have diverse responses to the same stimulus since they have specific synthesis and metabolism pathways. AEA and other NAEs are produced from a low abundance phospholipid, namely, N-acyl-phosphatidylethanolamine (NAPE)^50^, and are catabolized by hydrolysis of the amide bond through the actions of FAAH^48^ and N-acylethanolamine-hydrolysing acid amidase (NAAA), found primarily in peripheral tissues^51^. The relative proportion of the NAEs produced reflects the relative proportion of the acyl chains found in the sn-1 position of the donor phospholipids; therefore, the concentrations of AEA are commonly lower than that of PEA and OEA in human biological samples, like plasma and serum^14^, as confirmed in our samples. Hypothetically, we could say that the decrease in circulating NAEs 5 days and 12 days after surgery could reflect a common global response of these molecules to local skin injury. On the other hand, 2-AG might act as a different metabolic pathway: 2-AG is synthetized in cells that express diacylglycerol lipase, by activation of phospholipase C (PLC), and is catabolized by hydrolysis of its ester bond by several enzymes, such as alpha–beta hydrolase domain protein (ABHD)-6, ABHD-12 and monoacylglycerol lipase (MAGL)^52^. Regarding 2-AG, an increase in circulating concentrations was observed 5 days after surgery. This increase was also accompanied by extremely high variability between patients. These findings highlight that variance in 2-AG can be attributed to wound healing or surgery since most differences occurred immediately after surgical intervention. However, other common factors in patients undergoing hospitalization can cause 2-AG fluctuations, such as acute stress, anxiety or sleep disorders. It was shown in a previous study^53^ that 2-AG plasma concentrations increased significantly immediately after the beginning of cardiac surgery and reached maximal concentrations during cardiopulmonary bypass. However, in contrast to our results, after termination of cardiopulmonary bypass, 2-AG concentrations decreased significantly and were close to preoperative values at the time of admission in the cardiovascular intensive care unit. The authors suggest that the increase in 2-AG after initiation of cardiopulmonary bypass should be part of the inflammatory response. In our study, we were not able to collect plasma samples during surgery or immediately after the induction of general anaesthesia, but the 2-AG concentration in our study certainly remained elevated for at least 5 days after surgery. An inflammatory response is known to occur after surgery or skin injury^37^ and can also explain our results regarding 2-AG plasma fluctuation.

Several studies have reported that many personal characteristics, such as gender^54^, age^55^, BMI and the presence of metabolic dysfunction^56^, can influence circulating endocannabinoid concentrations. Food consumption and circadian rhythms also influence the endocannabinoid system^39,57^. Coincidentally, both of our groups (N and HT group) exhibited no significant differences in any of these features, and blood was collected on the same morning after an overnight fasting period for all patients. However, other physiologic and pathologic disorders, such as behavioural regulation of feeding^58^, psychiatric disorders such as anxiety and depression^59^ and fertility^60^, are involved in the endocannabinoid system. As no psychometric tests were performed before the study and fertility was not evaluated, these may have also contributed to the high variability in concentrations of plasma endocannabinoids observed between patients and may constitute a limitation to the study.

Regarding the relationship between circulating endocannabinoids and the skin, we found a positive correlation between the concentrations of AEA in plasma and in skin of patients who later developed normal scars. Curiously, this correlation was not present in patients who developed hypertrophic scars. Somehow it appears that regarding AEA, there is a link between both endocannabinoid systems (skin and systemic), and this link is lost in patients who develop hypertrophic scars. This was already observed before surgery. It should be noted that the number of patients included in the HT group (n = 10) was smaller than that included in the N group (n = 40). This may cause limitations in the interpretation of the lack of correlation in the HT group. It would be interesting to measure endocannabinoids and related NAEs in the skin during all wound-healing phases in all 50 patients to clarify the role of endocannabinoids. This would allow us not only to evaluate local variations during wound healing but also to understand whether this relation changes over time. However, this evaluation was not possible due to ethical implications, as it is not permitted to perform a surgery or inflict a wound in a patient for research purposes only. It remains to be established whether changes in peripheral concentrations reflect similar modifications in skin or if circulating changes may affect cutaneous functions, since these compounds, due to their lipophilicity, are believed to act as an autocrine/paracrine mediator^61^. Nevertheless, the present study demonstrates that in humans, AEA circulatory concentrations can reflect AEA concentration in the skin, and this is not true for PEA, OEA and 2-AG. It seems that AEA shares the same origin in both skin and systemic systems, in contrast to 2-AG, which appears to be under distinct local control.

In conclusion, female humans submitted to body-contouring surgery presented a time response pattern of plasma endocannabinoids and related NAEs, and the concentration of AEA in skin was positively correlated with the concentration of AEA in plasma. Patients who developed HT scars did not present this correlation, and AEA was significantly reduced in HT tissues compared to normal scar tissues. The current study adds to the available literature and increases knowledge on the role of the endocannabinoid system in wound healing and hypertrophic scarring of human skin.

Currently, patients frequently question their dermatologists about the effects of cannabis-derived products in the skin, but clinicians usually fail to find robust clinical evidence for their efficacy^62^. In fact, the data reported herein should certainly encourage researchers to further explore cannabinoid effects in human skin, namely, as an adjunct treatment strategy for hypertrophic scars or other wound-healing disorders.

## Material and methods

### Subjects

A prospective hospital-based study was conducted for 18 months. Fifty women submitted for routine body-contouring surgery (abdominoplasty, arm lift and thigh lift) in the Department of Plastic, Reconstructive and Aesthetic Surgery of Centro Hospitalar e Universitário de São João in Porto were selected. Exclusion criteria included additional surgeries 1 year before inclusion in this study, immunosuppressive therapy or postoperative complications.

Before surgery, all the subjects were asked to answer a survey concerning demographic data, alcohol, smoking and drug habits, medical and medication-use history, weight, height and history of past surgeries (including bariatric surgery).

Six months after surgery, patients were asked to attend a consultation to be evaluated by two independent trained plastic surgeons to decrease subjectivity. Scars were classified according to the Vancouver Scar Scale (VSS). Scars scoring ≥ 1 were classified as hypertrophic (HT group, n = 10), and scars scoring 0 were classified as normal scars (control group, N = 40)^63^.

### Blood and tissue samples

Blood samples of every subject included in the study were collected immediately before surgery (t0d), 5 days after surgery (t5d; corresponding to the inflammatory phase of wound healing), 12 days after surgery (t12d; corresponding to the proliferation phase of wound healing), and 3 months after surgery (t3m; corresponding to the remodelling phase of wound healing). All blood samples were taken in the morning after an overnight fasting state. Blood samples were collected by vein suction into a vacutainer containing EDTA. Phenylmethyl-sulfonyl-fluoride (PMSF) (100 μM final concentration), an inhibitor of fatty acid amide hydrolase (FAAH), was added to blood samples to prevent endocannabinoid and related NAEs degradation. Samples were then placed on ice and centrifuged within 1 h at 1,500 × g for 10 min at 4 °C. Plasma was removed to a fresh plastic tube and immediately stored at − 80 °C until processing and endocannabinoid analysis.

At time 0, skin samples (200 to 250 mg) taken from the abdomen, arm or thigh were surgically removed from skin flaps at the site of surgery. In detail, abdominal skin was collected from the left corner of the abdominal flap resected (left hypogastrium); arm skin was collected from the corner located near the elbow; and thigh skin was collected from the anterior corner in the resected inguinal flap. The subcutaneous fat was removed using surgical preparative scissors, and the skin was cut by a scalpel into pieces of 1 × 1 cm and immediately frozen in liquid nitrogen. Samples were stored at − 80 °C for posterior endocannabinoid and related NAEs quantification. Most bariatric patients undergo several body countering or revision surgeries. In total, of the 50 patients first included in the study, 12 underwent another surgery within the first postoperative year. During this second procedure, we were able to collect 12 scar samples from those patients (time 1, normal scars, n = 9 and hypertrophic scars, n = 3). Scars were collected and processed as described for skin samples.

### Endocannabinoid and related NAEs quantification

Anandamide, PEA, OEA and 2-AG were quantified in human plasma and skin for every collected sample using LC–MS/MS following extraction, as described below. All procedures were performed in the dark to protect the samples from degradation.

### Human skin sample extraction

Skin samples were thawed at 4 °C in ice. After weighing, 500 µL of phosphate buffer 0.1 mM pH 5.6 and 2 µL ISTD spiking solution containing AEA-d8, PEA-d4, OEA-d2 and 2AG-d8 (Cayman Chemical) were added to all samples. Chloroform:MeOH (2:1) 500 µL was added, and the samples were then vortexed vigorously for 2 cycles of 5 s at 5,000 rpm using a bead beater and centrifuged at 20,000 × g for 10 min at 4 °C, after which the organic layer was removed. This procedure was repeated three times, and all the organic phases were pooled. Then, the organic phase was evaporated in a CentriVap concentrator at 50 °C until dryness and reconstituted in 100 µL of acetonitrile. The supernatant was then transferred to HPLC vials to be injected (5 µL) into an LC–MS/MS device.

### AEA, PEA and OEA plasma sample extraction

Aliquots of human plasma (50.0 µL) were added to 400 µL of 1.0 µg/mL ISTD working solution containing AEA-d8, PEA-d4 and OEA-d2 in acetonitrile 0.1% formic acid for protein precipitation^64,65^. The samples were vortex-mixed and centrifuged for 10 min at 14,000 rpm at 4 °C, and the supernatant was injected (7 µL) into the LC–MS/MS machine.

### 2-AG plasma sample extraction

Aliquots of human plasma (500.0 µL) were added to 500 µL of internal standard working solution containing 10 ng/mL 2-AG-d8 in Milli-Q water. Samples placed into (16*125 mm) glass culture tubes were vortex-mixed and loaded (900 µL) into Oasis cartridges (HLB, 30 mg, 1 mL waters) previously conditioned with 1 mL of methanol and with 1 mL of water. After being loaded with the sample, the cartridges were washed twice with 0.5 mL of 40% aqueous methanol, and after the second wash, the cartridges were flushed with an air push of 2 mL at 1 mL/min. The samples were eluted twice with 1,000 µL of methanol with an air push of 2 mL at 1 mL/min. The eluate was placed under vacuum until reaching dryness for up to 2 h and then was reconstituted in 100 µL of acetonitrile. The samples were then injected (1 µL) into the LC–MS/MS device.

### LC–MS method

The analysis of sample extracts for AEA, PEA, OEA and 2-AG was performed using LC–MS/MS TQ (6,470, Triple Quad LC–MS Agilent Technologies, Santa Clara, California, EUA) with electrospray ionization and an Agilent jet stream. Separation for AEA, PEA and OEA was performed on an Agilent Poroshell 120 Phenyl-Hexyl, 4.6 × 50 mm; 2.7 µm, using water (A) and acetonitrile 0.1% formic acid (B) as the mobile phase and a stop time of 5 min. The separation for 2-AG was performed on a Waters XSelect CSH Phenyl-Hexyl, 3.5 µm, 4.6 × 50 mm column, using water 0.1% formic acid (A) and acetonitrile 0.1% formic acid (B) as the mobile phase and a stop time of 7 min. The flow rate was 0.5 mL/min, and samples were maintained at 4 °C throughout. The ionization mode was electrospray, polarity positive. Electrospray jetstream conditions were as follows: capillary voltage, 3,500 V; drying gas flow, 10 L/min nitrogen; drying gas temperature, 300 °C; nebulizer pressure, 30 psi; sheath gas temperature, 400 °C; and sheath gas flow, 11 L/min. The mass spectrometer was operated in the multiple reaction monitoring mode. The multiple reaction monitoring pair was m/z 326.5 → 62.1 for OEA; m/z 300.5 → 62.1 for PEA; m/z 328.5 → 62.1 for OEA-d2; m/z 304.5 → 62.1 for PEA-d4; m/z 348.3 → 62.1 for AEA; and m/z 356.6 → 62.1 for AEA-d8. The collision energy used for all compounds was 12 eV. For 2-AG, the multiple reaction monitoring pair was m/z 379.6 → 287.2 and m/z 387.6 → 294.3 for 2-AG-d8, with a collision energy of 14 eV. Peaks from standards and analyses were integrated using MassHunter Workstation software version B.04.00 (Agilent, Santa Clara, California, EUA), and the concentration of each compound was calculated using calibration curves of concentration against relative response. Together with the tissue samples, quality control (QC) samples were also extracted, evaporated and injected. A set of QC samples was placed at the beginning and at the end of the analytical run, demonstrating the good precision and accuracy of the overall process.

In plasma, the results are presented in pg/mL for AEA, PEA, OEA and 2-AG. The linearity ranged from 100 pg/mL to 10,000 pg/mL for AEA, PEA and OEA quantification and from 500 pg/mL to 50,000 pg/mL for 2-AG quantification. In human skin samples, the results are presented in pg/g, using a linear range from 100 pg/mL to 10,000 pg/mL for AEA quantification, from 200 pg/mL to 20,000 pg/mL for OEA and PEA quantification and from 1.0 ng/mL to 1,000 ng/mL for 2-AG quantification.

### Statistical analyses

The sample size was determined using G Power (Version 3.1). We considered a 30% effect size on the primary outcome (concentration of plasma AEA) to be clinically relevant and estimated a 20% standard deviation from previously published data^66^. Furthermore, we decided that the ratio between the control and experimental group size would be 4:1, according to the natural occurrence of the phenomenon. Using these parameters, we obtained a total sample size of 48 subjects for a 5% significance level and a statistical power of 90%.

The results are presented as the mean ± SEM. Analyses were carried out with Prism 7 (Version 7.0; GraphPad Software, Inc.). The Kolmogorov–Smirnov test was used to test for normal distribution. To analyse individual changes over time points in skin samples, Student’s paired t-test was used. To analyse differences between groups in skin samples, an unpaired t-test was used. Two-way ANOVA followed by Tukey’s multiple comparison test was used to compare concentrations of plasma endocannabinoids and related NAEs in each group. For correlation analyses, Pearson r was calculated with a 95% confidence value, and a linear regression was fitted for data concerning AEA plasma and skin data. *p* < 0.05 was considered significant.

### Statement of ethical approval

The study was approved by the Portuguese Institutional Review Board for Human Subjects (Comissão de Ética para a Saúde – Centro Hospitalar de São João) and carried out in accordance with principles of the Declaration of Helsinki as revised in 2001. All patients gave written informed consent to participate in this research.
